# P60. Microtubule-depolymerising agents used in antibody-drug-conjugates induce anti-tumour immunity by stimulation of dendritic cells

**DOI:** 10.1186/2051-1426-2-S2-P34

**Published:** 2014-03-12

**Authors:** K Martin, P Mueller, S Theurich, S Savic, G Terszowski, HM Kvasnicka, S Dirnhofer, DE Speiser, M von Bergwelt-Baildon, A Zippelius

**Affiliations:** 1 University Hospital Basel, Department of Biomedicine, Basel, Switzerland; 2 University Hospital Cologne, Department I of Internal Medicine and Cologne Interventional Immunology, Cologne, Germany; 3 University Hospital Basel, Institute of Pathology, Basel, Switzerland; 4 University of Frankfurt, Senckenberg Institute of Pathology, Frankfurt, Germany; 5 University of Lausanne, Ludwig Center for Cancer Research, Lausanne, Switzerland; 6 University Hospital Basel, Department of Biomedicine and Department of Medical Oncology, Basel, Switzerland

## 

Antibody drug conjugates (ADCs) are emerging as powerful treatment strategies with outstanding target specificity and high therapeutic activity in cancer patients. Brentuximab vedotin represents a first-in-class ADC directed against CD30-positive malignancies. We hypothesised that its sustained clinical responses could be related to the stimulation of an anti-cancer immune response. We here demonstrate that the dolastatin family of microtubule inhibitors, from which the cytotoxic component of brentuximab vedotin is derived, comprises potent inducers of phenotypic and functional DC maturation. In addition to the direct cytotoxic effect on tumour cells, dolastatins efficiently promoted antigen uptake and migration of tumour-resident DCs to tumour-draining lymph nodes. Exposure of murine and human DCs to dolastatins significantly increased their capacity to prime T cells. Underlining the requirement of an intact host immune system for the full therapeutic benefit of dolastatins, the anti-tumour effect was far less pronounced in immune-compromised mice. When combining dolastatins with tumour-antigen-specific vaccination or blockade of the PD-1/PD-L1 and CTLA-4 co-inhibitory pathways, we observed substantial therapeutic synergies. Ultimately, ADCs using dolastatins induce DC homing and activate cellular anti-tumour immune responses in patients. Our data reveal a novel mechanism of action for dolastatins and provide a strong rationale for clinical treatment regimens combining dolastatin-based therapies, such as brentuximab vedotin, with immune-based therapies.

